# OPAL: a randomised, placebo-controlled trial of opioid analgesia for the reduction of pain severity in people with acute spinal pain—a statistical analysis plan

**DOI:** 10.1186/s13063-022-06028-y

**Published:** 2022-03-14

**Authors:** Caitlin MP Jones, Chung-Wei Christine Lin, Richard O. Day, Bart W. Koes, Jane Latimer, Chris G. Maher, Andrew McLachlan, Laurent Billot

**Affiliations:** 1 Institute for Musculoskeletal Health, Sydney Local Health District and The University of Sydney, Level 10N, KGV Building, Missenden Road, Camperdown, Sydney, New South Wales Australia; 2 Faculty of Pharmacy and The Centre for Education and Research on Ageing (CERA), The University of Sydney and Concord Hospital, Sydney, New South Wales Australia; 3 Department of Clinical Pharmacology & Toxicology, St Vincent’s Hospital Sydney and St Vincent’s Clinical School, Faculty of Medicine, University of New South Wales, Sydney, New South Wales Australia; 4 Department of General Practice, Erasmus MC, Rotterdam, The Netherlands; 5 The George Institute for Global Health, Faculty of Medicine, University of New South Wales, Sydney, Australia

**Keywords:** Opioids, Low back pain, Neck pain, Placebo-controlled trial, Statistical analysis plan

## Abstract

**Background:**

Low back and neck pain are a leading cause of disease burden globally. Opioids are recommended in guidelines for acute low back and neck pain; however, there is a lack of compelling efficacy data to support this.

**Methods:**

The OPAL trial is a prospectively registered, triple-blinded, randomised, placebo-controlled trial. Patients with acute (≤12 weeks duration) back and/or neck pain receive guideline care plus either an opioid (oxycodone + naloxone, up to 20 mg per day) or a placebo for up to 6 weeks or earlier, if pain is resolved. The primary outcome is pain measured using the Pain Severity Score of the Brief Pain Inventory with the primary time point being 6 weeks. Secondary outcomes include physical function, time to recovery, quality of life, adverse events and risk of opioid misuse. Outcomes are collected at weeks 2, 4, 6, 12, 26 and 52. Analysis will be done on an intention-to-treat principle. *p* values of < 0.05 will be considered significant and 95% confidence intervals will be reported. Repeated-measures linear mixed models will be used to assess the effect of the treatment group on the primary outcome and continuous secondary outcomes. Adverse events will be compared between groups using Fisher’s exact test. Cost-effectiveness analyses will be conducted if a treatment effect on pain is seen at week 6. Subgroup analyses will be performed to assess whether pain duration and pain location are treatment effect modifiers.

**Discussion:**

The OPAL trial will provide important evidence about whether a short course of opioids is effective in the treatment of acute non-specific low back and/or neck pain. This pre-specified statistical analysis plan details the methodology for the analysis of the OPAL trial results.

**Trial registration:**

ACTRN12615000775516. The trial has completed recruitment. Follow-up on the last patient will be completed in March 2022.

## Background

Low back pain and neck pain are extremely prevalent and are leading causes of disease burden globally [1]. Strong analgesics, such as opioid analgesics, are recommended by clinical guidelines for people with acute low back pain or neck pain who require more pain relief [2]. Opioid analgesics are widely and increasingly used [3], but there is a lack of compelling efficacy data supporting the use of opioid analgesics for acute low back pain or neck pain [4]. A placebo-controlled trial of opioids for acute non-specific spinal pain has not previously been conducted; therefore, we cannot be certain what benefits opioids might provide. Concerns regarding opioid use are further heightened by the risks of adverse events, some of which can be serious (e.g. dependency, misuse and overdose) [5].

The OPAL study is the world’s first randomised placebo-controlled trial which is investigating the efficacy and safety of opioid analgesics in people with acute low back and/or neck pain [6]. Three-hundred and forty-seven participants are randomised to receive either the study medication (modified release oxycodone up to 20 mg per day) or a placebo for up to 6 weeks in addition to guideline-recommended care. The primary outcome is pain severity at 6 weeks. Other outcomes include pain severity, physical functioning, time to recovery, quality of life (physical and mental subscales) and occurrence of adverse events, measured at regular time points up to 12 months.

## Methods/design

### Study objectives

The primary objective is to investigate differences in pain severity at 6 weeks between participants randomised to the opioid arm and those randomised to placebo. We hypothesise that participants randomised to treatment with an opioid analgesic will have lower pain severity. The null hypothesis is that there is no difference in mean pain severity score between the two arms at 6 weeks.

Secondary objectives are to investigate whether the opioid analgesic:

- Reduces pain at 12 weeks
- Improves disability, time to recovery, quality of life (physical and mental) and global improvement over the 6-week treatment period and at 12 weeks
- Is tolerated
- Is cost-effective from the health system and societal perspectives
- Does not result in opioid analgesic misuse at 3, 6 and 12 months

### Patient population

Participants are recruited as they present to general practitioners (GP) or a hospital emergency department with a primary complaint of low back and/or neck pain. A doctor screens the patient against the inclusion and exclusion criteria. If they are eligible, the doctor seeks informed consent prior to enrolling them into the study.

Participants in the community experiencing acute back and/or neck pain can also be identified via advertisements on social media. Potential participants respond to the social media advertisement by completing an online pre-screening questionnaire and are encouraged to contact the OPAL trial team if they meet the pre-screening criteria. They are then referred to a study GP for further screening.

### Inclusion criteria

- Low back pain (pain between the 12th rib and buttock crease) and/or neck pain (pain in the area below the occiput to the most distal cervical spine) with or without distal radiation to the leg (for low back pain) or arm (for neck pain)
- The current episode of pain is no more than 12 weeks since onset and preceded by at least a 1-month pain-free period (to screen out those with persistent pain)
- Pain severity is at least moderate (as measured by adaptations of item 7 of the SF-36, i.e. how much low back pain or neck pain have you had in the last week? *None/very mild/mild/moderate/severe/very severe*).

### Exclusion criteria

- Known or suspected serious spinal pathology (e.g. infection, cauda equina syndrome, spinal fracture)
- Contraindications to opioid analgesics (including previous intolerance to opioids or adverse reaction, opioid addiction history, allergy to any ingredient in the opioid or placebo tablets, prior or current abuse of psychoactive drugs, prior or current alcoholism) or scoring ‘high risk’ (a score of 9 or above) on the Opioid Risk Tool
- Have taken a prescription opioid analgesic for the current episode of low back pain and/or neck pain at a dose > 15 mg of oral morphine equivalent per day for 5 or more consecutive days
- Spinal surgery in the preceding 6 months
- Scheduled or being considered for surgery or interventional procedures for low back pain and/or neck pain during the 6-week treatment period
- Less than 18 years of age
- Not having sufficient English language skills to understand trial procedures or complete assessments, or suitable translation is not available
- For female participants: planning conception or are pregnant or breastfeeding

There were changes to the exclusion criteria during the trial to facilitate recruitment and in response to the up-scheduling of codeine from over-the-counter to a prescription-only medicine in Australia in February 2018. The original protocol (version 1.0) states that participants must have had back and/or neck pain for a minimum of 2 weeks and excluded those who have taken any prescription opioid for the current episode of back and/or neck pain.

### Outcomes

**Table:** 

**Outcome**	**Measurement tool**	**Measurement occasions**
***Primary outcome***		
Pain severity	Pain Severity Score of the Brief Pain Inventory	Baseline, weeks 2, 4, 6 and 12
***Secondary outcomes***		
Physical functioning (generic)	Pain Interference Score of the Brief Pain Inventory.	Baseline, weeks 2, 4, 6 and 12
Physical functioning (condition-specific)	Roland-Morris Disability Questionnaire (24 items, for participants reporting low back pain only) and Neck Disability Questionnaire (for participants reporting neck pain only)	Baseline and 6 weeks
Time to recovery (average daily pain of 0 or 1 of 10 for the past seven consecutive days)	Pain diary	Daily until recovery or up to 12 weeks
Quality of life (physical)	SF-12	Baseline, weeks 2, 4, 6 and 12
Quality of life (mental)	SF-12	Baseline, weeks 2, 4, 6 and 12
Participants’ rating of global improvement	Global Perceived Effect scale	Baseline, weeks 2, 4, 6 and 12
***Other outcomes***		
Adverse events	Self-report and doctor report	Self-report at weeks 2, 4, 6 and 12 Doctor report after each follow-up visit
Work absenteeism	Self-report	Baseline, weeks 2, 4, 6 and 12
Use of treatment or health care services	Self-report	Baseline, weeks 2, 4, 6 and 12
Compliance to study medication	Self-reported adherence recorded in a medication diary compared against doctor prescription data, supported by returned medicine count	Medicine diary is recorded daily over 6 weeks Doctor prescription data at each visit Returned medicine count at end of treatment period (≤6 weeks)
Success of blinding	Participants are asked to estimate their allocation group as active opioid, inactive placebo or do not know	Week 6
***Long-term outcomes***		
Pain severity	Pain Severity Score of the Brief Pain Inventory	Weeks 26 and 52
Use of treatment or health care services	Self-report	Weeks 26 and 52 if still experiencing low back pain and/or neck pain (> 1/10)
Risk of misuse	Current Opioid Misuse Measure	Weeks 12, 26 and 52

### Intervention

Participants randomised to the treatment group receive modified release oxycodone plus naloxone up to 20 mg per day for up to 6 weeks. Participants randomised to the placebo group receive identical placebo tablets. Both groups also receive guideline care (reassurance of a positive prognosis, advice to stay active and avoid bed rest and, if required, other guideline-recommended treatments) [2].

The medication regimen starts at a dose of 5 mg oxycodone/naloxone, 2 times a day. This is gradually titrated up to the maximum dose of 10 mg, 2 times a day based on individual participant progress, tolerability and sedation score, before down titration to cessation. Treatment will continue until ‘adequate improvement’ (0 to 1 out of 10 pain for 3 consecutive days) or for a maximum of 6 weeks. For participants screened by a trial GP, their treatment is provided and monitored by the GP. For participants screened at the emergency department, their treatment is provided and monitored by a hospital rheumatologist.

### Randomisation and blinding

An a priori allocation sequence was prepared by an independent statistician. Trial medication packs were prepared according to the sequence and were prepared and dispatched to trial pharmacies by an independent supplier. Upon recruitment, the participant is allocated the next sequentially numbered medication pack, randomising the participant on a 1:1 ratio. Blinding of the trial doctors, pharmacist, participant, assessor and data analyst is ensured by randomisation and concealed allocation. Blinding will be maintained until all data have been collected and analysis and interpretation have been completed and agreed upon. The success of participant blinding will be tested at 6 weeks.

### Sample size

A sample size of 173 per group (346 total) will be sufficient to detect a clinically meaningful between-group difference of 1 on a 10-point pain scale at 6 weeks, assuming a *SD* of 2.5, power of 90% and *α* of 5% and allowing for 5% dropout and 10% non-compliance. No studies have reported on a minimum clinically important difference for acute spinal pain, but we expect patients would need to perceive at least one point of pain reduction to consider the treatment worthwhile.

## Statistical analysis

### General principles

This analysis plan applies to all data collected during the OPAL trial. Analyses will be conducted using SAS Enterprise Guide version 8.3 or above (SAS Institute Inc. 2012). Analyses will be conducted by the trial team and by an independent biostatistician using randomly permuted (‘scrambled’) treatment allocations. Real treatment allocations will be used once the validation is completed and the statistical code finalised. Data interpretation will first be performed using the real but masked treatment allocation.

### Confidence intervals and *p* values

A *p* value of < 0.05 will be considered statistically significant. 95% confidence intervals will be reported to aid interpretation of precision and clinical importance of the findings.

### Timing of final analysis

All outcomes will be analysed collectively at the completion of participant recruitment and following up. Recruitment was completed in March 2021 (347/346 participants recruited). The expected date of the final visit is March 2022 (approximately 12 months after completion of recruitment).

### Interim analysis

A data safety and monitoring committee met on 2 occasions and oversaw interim pain intensity and adverse event outcomes during the conduct of the trial. No formal interim efficacy analysis was conducted.

### Multiplicity adjustment

Given the clear outcome hierarchy and limited number of related secondary outcomes, we do not plan to adjust for multiplicity.

### Data sets to be analysed

The intention-to-treat (ITT) population is all participants randomised regardless of compliance to study treatment. Unless otherwise specified, all analyses will be conducted on an intention-to-treat basis.

### Subject disposition

**Fig. 1:**
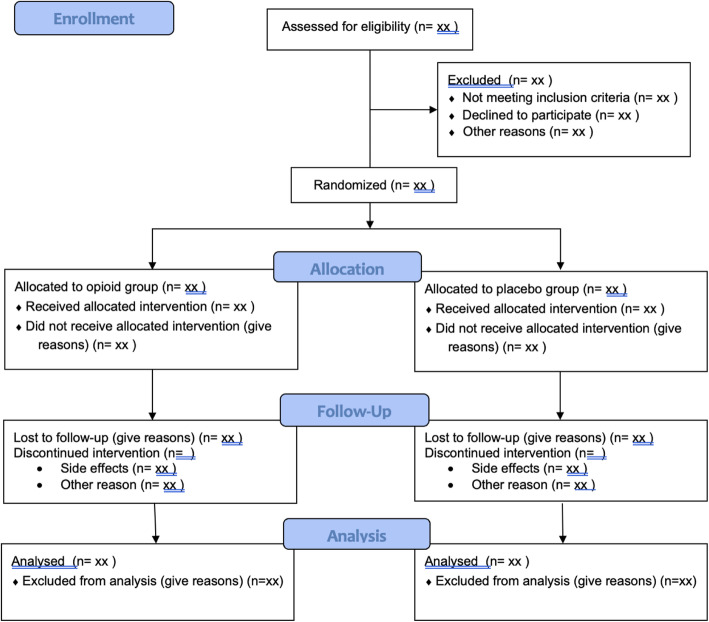
Consort 2010 flow diagram

The flow of participants through the trial including the number of withdrawals and participants lost to follow-up will be presented in a CONSORT flow diagram (see Fig. 1 in the Appendix). Timing and reasons for withdrawal and lost to follow-up will also be presented in the flow diagram.

### Participant characteristics and baseline comparisons

Baseline participant characteristics will be presented in a table (see Table 1 in the Appendix). Discrete variables will be summarised by frequencies and percentages. Percentages will be calculated according to the number of participants for whom data are available. Continuous variables will be summarised using mean and SD, and median and interquartile range (Q1–Q3). We do not intend to perform statistical tests comparing groups on the baseline data. Baseline characteristics collected are:

- Sex
- Age
- Body mass index (kg/m^2^)
- Location of pain (back/neck/both)
- Days since onset of pain
- Number of previous episodes of back and/or neck pain
- Employment status
- Employment classification
- Whether pain is compensable
- Household weekly income
- Health insurance status
- Pain severity
- Disability (generic- and condition-specific)
- Quality of life (physical and mental subscales)
- Global perceived effect since onset of pain
- Healthcare utilisation prior to enrolment, including use of opioid medications

### Compliance to the study intervention

Compliance data will be displayed as per Table 2 in the Appendix. Compliance is determined primarily by looking at the participant’s medication reported in the daily clinical trial medicines diary when compared to the doctor’s prescription (and supply) of clinical trial medicines. Acceptable compliance will be defined as consuming ≥80% of their prescribed clinical trial medication. Secondarily, we will also look at the returned medicine count to confirm the participant’s self-reported medication diary data. Compliance with the randomised intervention will be summarised using the following variables:

- Number of treatment discontinuations
- Discontinuation reasons (where available)
- Daily dose of medication taken
- Cumulative dose of medication taken over the course of the study
- Compliance, defined as taking at least 80% of prescribed medication

Protocol deviations are defined as any deviations from the study protocol (1), including eligibility criteria, concomitant medications or study conduct. A list of deviations will be presented in a table including the participant ID, date of deviation, details of the deviation, classification of deviation, whether the data was excluded from analysis and whether the participant needed to be replaced. See protocol deviations in Table 3 in the Appendix.

### Concomitant therapies

Type and frequency of concomitant interventions will be categorised and presented as per Table 5 in the Appendix.

### Analysis of the primary outcome

Descriptive analysis will consist of raw mean scores at each visit (baseline, weeks 2, 4 and 6 and months 3, 6 and 12) (see Table 4 in the Appendix). Raw data will also be reported on a longitudinal mean plot together with 95% confidence intervals.

### Main analysis

Repeated-measure linear mixed models will be used to assess the effect of the treatment group on pain severity. The model will include outcome data collected at every post-baseline visit, i.e. at weeks 2, 4, 6, 12, 26 and 52. Fixed effects will include the randomised treatment allocation, the follow-up time points as a categorical variable with 6 levels, the interaction between treatment and time point as well as the baseline pain severity score.

Correlations between repeated measures (weeks 2, 4, 6, 12, 26 and 52 assessments) will be modelled using a repeated effect with a compound-symmetry structure. The primary treatment effect will be estimated as the adjusted mean difference in pain severity at the week 6 visit between the opioid and placebo arms together with its 95% CI.

The same model will be used to estimate the effect of the treatment at weeks 12 and 52.

### Adjusted analysis

We will conduct sensitivity analyses after adding duration of the current pain episode and site of pain, that is, low back or neck, as covariates to the main model. This will be done both on the non-imputed and imputed (if applicable) data.

### Subgroup analyses

The primary analysis described under the ‘main analysis’ heading will be repeated according to the site of pain (low back or neck). We will consider sex (male or female) as an additional subgroup. We anticipate that this will be reported in an appendix of the main report or in a separate manuscript.

This will be done by adding the subgroup variable to the main model together with its interaction with the randomised treatment, with the visit and with the treatment by visit interaction.

Heterogeneity by subgroup will be assessed by estimating the effect of the intervention at week 6 within each subgroup as well as through the corresponding interaction *p* value (at week 6 only). The results will be presented on a forest plot.

In addition, heterogeneity of treatment effect according to pain duration will be assessed using pain duration as a continuous variable.

### Complier average causal effect

In case of substantial departure from compliance (see definition above), we will estimate the effect of the intervention in compliers using a Complier Average Causal Effect (CACE) with a propensity score and/or a joint-modelling approach. Details of the approach will be defined after reviewing the main results. The CACE analysis will therefore be considered exploratory.

### Treatment of missing data

In case > 10% of the primary outcome data at week 6 are missing, multiple imputations will be used to conduct sensitivity analyses for the longitudinal linear mixed model of the primary outcome. The imputation model will include the treatment arm, all baseline variables (listed above) and all the pain severity score, the physical functioning scores, the quality of life scores and the global improvement score collected at baseline and all follow-up visits. One hundred sets will be imputed using fully conditional specification with discrete variables imputed using a discriminant function [7]. Each imputed set of data will be analysed using the same model as the one used for the main analysis, both without and with adjustment for additional covariates (see the ‘Adjusted analysis’ section). Subgroup analyses will not be conducted on the imputed data set.

### Analysis of secondary outcomes

Results will be presented as per Table 4 in the Appendix.

### Continuous secondary outcomes

Continuous secondary outcomes include the following scores:

- Disability measured by the Pain Interference Score
- Disability measured by the Roland-Morris Disability score
- Disability measured by the Neck Disability Questionnaire score
- SF-12 scores
- Participants’ rating of global improvement

All will be described using a longitudinal mean plot and analysed using repeated-measure linear mixed models mirroring the approach used for the analysis of the primary outcome to estimate the effect of the treatment at weeks 6 and 12.

The same imputed datasets as the one created in the imputation of the primary outcome will be used to repeat the analysis of these five continuous secondary outcomes. Multiple imputations will only be performed if the primary outcome at week 6 is missing for more than 10% of subjects.

### Time to recovery

Daily pain data collected from the patient diary will be described using a heatmap. The heatmap will consist of one colour-coded (by pain intensity) square per day (*x*-axis) per patient (*y*-axis) and two side-by-side panels (opioid vs placebo) up to week 6.

Time to recovery is defined as an average daily pain of 0 or 1 (out of 10) for seven consecutive days). The day of ‘event’ will be the first day of the recovery, i.e. day 1 of the seven consecutive days. In the absence of event, data will be censored at 12 weeks (day 84) or at the point when no more daily data is collected, whichever is sooner.

Time to recovery will be described using a Kaplan-Meier plot and summarised as the median number of days to recovery together with quartiles (if available). Differences in survival curves will be assessed using the log-rank test (see Table 4 in the Appendix).

Additionally, we will compare the proportion of participants who have recovered between groups (up to week 12) as a dichotomous measure to support the recovery outcome.

### Healthcare utilisation

We will describe the type and frequency of services and medications used and assess between-group differences using Fisher’s exact test. We will also report what proportion of participants in each group (opioid and placebo) are still experiencing low back pain or neck pain at 26 and 52 weeks and the use of healthcare services, including opioid analgesics, in these participants. Differences in proportions will be tested using Fisher’s exact test (see Table 5 in the Appendix).

### Risk of misuse

We will report the proportion in each group deemed to be at risk of misuse as per the COMM risk of misuse screening tool (scoring ≥9 indicates the risk of misuse) at weeks 12, 26 and 52. Differences in proportions will be tested using Fisher’s exact test (see Table 4 in the Appendix).

### Cost-effectiveness

Two analyses will be conducted if between-group differences are found in the primary analysis: (1) a cost-effectiveness analysis using pain severity as a measure of effectiveness and (2) a cost-utility analysis where health state utilities (quality-adjusted life years or QALY) will be based on measures obtained from the SF-12 and transformed into utilities via the SF-6D algorithm. These analyses will not be included in the main paper and will be refined further following the main results.

The primary analysis will be conducted from the health sector’s perspective to assess the incremental cost per 1-point pain reduction or per QALY gained between the two treatment groups over 12 weeks.

A secondary analysis will entail a societal perspective in which costs associated with the use of community services (e.g. exercise classes) and work absenteeism related to low back pain or neck pain will be included. Costs of community services will be based on the self-reported costs. Costs of absenteeism from paid employment will be estimated by the number of days absent from work multiplied by the average wage rate. Sensitivity analyses will test uncertainty in key parameters such as the selection of cost weights and statistical variation in quality-of-life scores.

To obtain costs, public health services will be valued at published standard rates (e.g. the Medical Benefits Scheme standard fees, the Pharmaceutical Benefits Scheme costs). Private non-medical health services (e.g. physiotherapy) will be valued at published standard rates, if available, or as reported by participants.

### Analysis of safety outcomes

Between-group analysis of safety outcomes will be presented as per Table 5 in the Appendix.

### Adverse events

All adverse events are reported per ICD-10 code to level 3. Serious adverse events (see definition below) are also categorised for relatedness (yes/no) and expectedness (yes/no). The proportion of participants with adverse events overall and within each category (serious and nonserious) will be compared between the two groups using Fisher’s exact test. Self-reported data will be used as the primary source of data and supported by data reported by the doctor. Where participants and doctors have reported the same AE on the same dates, we will exclude the doctor data to avoid duplication. Please see Table 5 in the Appendix for aggregate data and Table 6 in the Appendix for detailed adverse events and serious adverse events.

Serious adverse events are any untoward medical occurrence that [8]

- Results in death
- Is life-threatening
- Requires hospitalisation or prolongation of existing hospitalisation
- Results in persistent or significant disability or incapacity
- Is a congenital anomaly or birth defect or
- Is a medically significant or important event or reaction

## Conclusion

The OPAL trial will be the world’s first placebo-controlled trial of opioids for acute non-specific spinal pain and aims to provide high-quality evidence on the efficacy of a short course of opioid analgesics for the treatment of acute, non-specific low back and/or neck pain. The results of the OPAL trial will provide much needed evidence to clinicians and is likely to influence international clinical guidelines. The findings of the OPAL trial will impact the healthcare system and society by helping to ensure that opioids are only being used where the benefits outweigh the harms. The planned statistical analysis is detailed here to provide transparency.

## Data Availability

All investigators have access to the study data. Raw data (de-identified) can be provided upon request.

## Appendix: Planned tables and figures

**Table 1:** Baseline characteristics

	Opioid (*n* = xxx)	Placebo (*n* = xxx)
Female	*n*/*N* (%)	*n*/*N* (%)
Age (years)	xx.x (SD), *n*	xx.x (SD), *n*
BMI (kg/m^2^)	xx.x (SD), *n*	xx.x (SD), *n*
Location of pain		
Back	*n*/*N* (%)	*n*/*N* (%)
Neck	*n*/*N* (%)	*n*/*N* (%)
Both	*n*/*N* (%)	*n*/*N* (%)
Days since onset of pain	Med (IQR)	Med (IQR)
Number of previous episodes of pain	Med (IQR)	Med (IQR)
Currently employed	*n*/*N* (%)	*n*/*N* (%)
Employment classification	*n*/*N* (%)	*n*/*N* (%)
Manager	*n*/*N* (%)	*n*/*N* (%)
Technician and trade worker	*n*/*N* (%)	*n*/*N* (%)
Clerical and administrative	*n*/*N* (%)	*n*/*N* (%)
Machinery operator or driver	*n*/*N* (%)	*n*/*N* (%)
Professional	*n*/*N* (%)	*n*/*N* (%)
Community or personal services worker	*n*/*N* (%)	*n*/*N* (%)
Sales worker	*n*/*N* (%)	*n*/*N* (%)
Labourer	*n*/*N* (%)	*n*/*N* (%)
Compensable back/neck pain	*n*/*N* (%)	*n*/*N* (%)
Household income/week (years) (AUD)		
No income	*n*/*N* (%)	*n*/*N* (%)
$1–$799 ($1–$41,599)	*n*/*N* (%)	*n*/*N* (%)
$800–$1999 ($41,600–$103,999)	*n*/*N* (%)	*n*/*N* (%)
$2000–$3999 ($104,000–$207,999)	*n*/*N* (%)	*n*/*N* (%)
$4000 or more ($208,000 or more)	*n*/*N* (%)	*n*/*N* (%)
Chose not to answer	*n*/*N* (%)	*n*/*N* (%)
Health insurance status		
None	*n*/*N* (%)	*n*/*N* (%)
Private hospital only	*n*/*N* (%)	*n*/*N* (%)
Private extras only	*n*/*N* (%)	*n*/*N* (%)
Private hospital and extras	*n*/*N* (%)	*n*/*N* (%)
DVA	*n*/*N* (%)	*n*/*N* (%)
Chose not to answer	*n*/*N* (%)	*n*/*N* (%)
Pain severity (BPI pain severity subscale average) (x/10)	Mean (SD)	Mean (SD)
Disability (BPI pain interference subscale average) (x/10)	Mean (SD)	Mean (SD)
Condition-specific disability		
Back – RMDQ (0–24)	*N* = mean (SD)	*N* = mean (SD)
Neck – NDI (0–50)	*N* = mean (SD)	*N* = mean (SD)
Quality of life – Physical SF12v2	Mean (SD)	Mean (SD)
Quality of life – Mental SF12v2	Mean (SD)	Mean (SD)
Global perceived effect scale (−5 to 5)	Mean (SD)	Mean (SD)
Healthcare utilisation prior to enrolment		
Took prescription opioid medication for back/neck pain	*n*/*N* (%)	*n*/*N* (%)
Saw a physiotherapist	*n*/*N* (%)	*n*/*N* (%)
Had imaging	*n*/*N* (%)	*n*/*N* (%)
Other healthcare	*n*/*N* (%)	*n*/*N* (%)
On medication for other pain or mood, sleeping, seizures or mental health problems during this episode of pain	*n*/*N* (%)	*n*/*N* (%)

*BPI* Brief Pain Inventory, *RMDQ* Roland-Morris Disability Questionnaire, *NDI* Neck Disability Index, *SF12v2* Short form 12, version 2

**Table 2:** Compliance

	Opioid (*n* = xxx)	Placebo (*n* = xxx)
GP prescription daily dose of study medication — either opioid or placebo (mg/day)	Mean (95% CI), *n*	Mean (95% CI), *n*
Week 1	xx.x (xx.x to xx.x), *n*	xx.x (xx.x to xx.x), *n*
Week 2	xx.x (xx.x to xx.x), *n*	xx.x (xx.x to xx.x), *n*
Week 3	xx.x (xx.x to xx.x), *n*	xx.x (xx.x to xx.x), *n*
Week 4	xx.x (xx.x to xx.x), *n*	xx.x (xx.x to xx.x), *n*
Week 5	xx.x (xx.x to xx.x), *n*	xx.x (xx.x to xx.x), *n*
Week 6	xx.x (xx.x to xx.x), *n*	xx.x (xx.x to xx.x), *n*
Cumulative dose (over entire treatment period)	xxx mgs	xxx mgs
Self-reported daily dose (mg/day)	Mean (95% CI), *n*	Mean (95% CI), *n*
Week 1	xx.x (xx.x to xx.x), *n*	xx.x (xx.x to xx.x), *n*
Week 2	xx.x (xx.x to xx.x), *n*	xx.x (xx.x to xx.x), *n*
Week 3	xx.x (xx.x to xx.x), *n*	xx.x (xx.x to xx.x), *n*
Week 4	xx.x (xx.x to xx.x), *n*	xx.x (xx.x to xx.x), *n*
Week 5	xx.x (xx.x to xx.x), *n*	xx.x (xx.x to xx.x), *n*
Week 6	xx.x (xx.x to xx.x), *n*	xx.x (xx.x to xx.x), *n*
Cumulative dose	xxx mgs	xxx mgs
Participants returning study medicines	*n*/*N* (%)	*n*/*N* (%)
Participants consuming ≥80% of prescribed dose		
Per participant medicine dairy	*n*/*N* (%)	*n*/*N* (%)
Per returned medicines	*n*/*N* (%)	*n*/*N* (%)
Participants discontinuing study medication		
	*n*/*N* (%)	*n*/*N* (%)
Reasons for discontinuation		
Pain resolved	*n*/*N* (%)	*n*/*N* (%)
Felt medicine was ineffective	*n*/*N* (%)	*n*/*N* (%)
Others	*n*/*N* (%)	*n*/*N* (%)
Assessment of participant blinding (participant estimation)		
Opioid	*n*/*N* (%)	*n*/*N* (%)
Placebo	*n*/*N* (%)	*n*/*N* (%)
Do not know	*n*/*N* (%)	*n*/*N* (%)

*mg/day* milligrammes per day

**Table 3:** Protocol deviations

Classification	Opioid	Placebo
Became apparent post-randomisation that participant did not fulfil eligibility criteria	*n*/*N* (%)	*n*/*N* (%)
Took opioid medicines for this episode	*n*/*N* (%)	*n*/*N* (%)
Pain duration > 12 weeks	*n*/*N* (%)	*n*/*N* (%)
Had bony metastasis	*n*/*N* (%)	*n*/*N* (%)
Did not receive treatment as allocated	*n*/*N* (%)	*n*/*N* (%)
Took concomitant opioid medication during the treatment period	*n*/*N* (%)	*n*/*N* (%)
Never collected medication kit	*n*/*N* (%)	*n*/*N* (%)
Was dispensed medicine from an incorrect kit	*n*/*N* (%)	*n*/*N* (%)
Discrepency in data collection procedure	*n*/*N* (%)	*n*/*N* (%)
Verbal consent received prior to enrolment, written consent followed	*n*/*N* (%)	*n*/*N* (%)
Unable to obtain baseline data	*n*/*N* (%)	*n*/*N* (%)
Obtained Brief Pain Inventory from a proxy	*n*/*N* (%)	*n*/*N* (%)
Baseline questionnaire completed > 72 h after first presentation to study doctor	*n*/*N* (%)	*n*/*N* (%)

**Table 4:** Primary and secondary outcomes

	Opioid (*n* = xxx)	Placebo (*n* = xxx)	Mean difference (95% *CI*), *p* value
Pain Intensity (BPI-PS)			
Week 2	xx.x (xx.x), *n*	xx.x (xx.x), *n*	
Week 4	xx.x (xx.x), *n*	xx.x (xx.x), *n*	
Week 6	xx.x (xx.x), *n*	xx.x (xx.x), *n*	xx.x (xx.x to xx.x), *p* = 0.xxx
Week 12	xx.x (xx.x), *n*	xx.x (xx.x), *n*	xx.x (xx.x to xx.x), *p* = 0.xxx
Week 26	xx.x (xx.x), *n*	xx.x (xx.x), *n*	
Week 52	xx.x (xx.x), *n*	xx.x (xx.x), *n*	xx.x (xx.x to xx.x), *p* = 0.xxx
Physical functioning - generic (BPI-IS)			
Week 2	xx.x (xx.x), *n*	xx.x (xx.x), *n*	
Week 4	xx.x (xx.x), *n*	xx.x (xx.x), *n*	
Week 6	xx.x (xx.x), *n*	xx.x (xx.x), *n*	xx.x (xx.x to xx.x), *p* = 0.xxx
Week 12	xx.x (xx.x), *n*	xx.x (xx.x), *n*	xx.x (xx.x to xx.x), *p* = 0.xxx
Physical functioning - back (RMDQ)			
Week 6	xx.x (xx.x), *n*	xx.x (xx.x), *n*	xx.x (xx.x to xx.x), *p* = 0.xxx
Physical functioning – neck (NDI)			
Week 6	xx.x (xx.x), *n*	xx.x (xx.x), *n*	xx.x (xx.x to xx.x), *p* = 0.xxx
Quality of life – physical score (SF-12v2)			
Week 2	xx.x (xx.x), *n*	xx.x (xx.x), *n*	
Week 4	xx.x (xx.x), *n*	xx.x (xx.x), *n*	
Week 6	xx.x (xx.x), *n*	xx.x (xx.x), *n*	xx.x (xx.x to xx.x), *p* = 0.xxx
Week 12	xx.x (xx.x), *n*	xx.x (xx.x), *n*	xx.x (xx.x to xx.x), *p* = 0.xxx
Quality of life – mental score (SF-12v2)			
Week 2	xx.x (xx.x), *n*	xx.x (xx.x), *n*	
Week 4	xx.x (xx.x), *n*	xx.x (xx.x), *n*	
Week 6	xx.x (xx.x), *n*	xx.x (xx.x), *n*	xx.x (xx.x to xx.x), *p* = 0.xxx
Week 12	xx.x (xx.x), *n*	xx.x (xx.x), *n*	xx.x (xx.x to xx.x), *p* = 0.xxx
Global perceived effect scale			
Week 2	xx.x (xx.x), *n*	xx.x (xx.x), *n*	
Week 4	xx.x (xx.x), *n*	xx.x (xx.x), *n*	
Week 6	xx.x (xx.x), *n*	xx.x (xx.x), *n*	xx.x (xx.x to xx.x), *p* = 0.xxx
Week 12	xx.x (xx.x), *n*	xx.x (xx.x), *n*	xx.x (xx.x to xx.x), *p* = 0.xxx
Time to recovery (days)	Median (IQR)	Median (IQR)	Log-rank *p* = 0.xxx
Recovered (yes)			
Week 2	*n* (%)	*n* (%)	
Week 4	*n* (%)	*n* (%)	
Week 6	*n* (%)	*n* (%)	*p* = 0.xxx
Week 12	*n* (%)	*n* (%)	*p* = 0.xxx

*BPI-PS* Brief Pain Inventory Pain Severity, *BPI-IS* Brief Pain Inventory Interference Subscale, *RMDQ* Roland-Morris Disability Questionnaire, *NDI* Neck Disability Index, *SF-12* Short Form 12 Item Survey

Note: We also intend to include similar looking tables for adjusted analyses and for analyses of imputed data as supplements

**Table 5:** Safety and other outcomes

	Opioid (*n* = xxx)	Placebo (*n* = xxx)	Fisher exact *p* value
Safety			
Serious adverse events (SAEs)	*n*^EVT^ *n*^PAT^ (%)	*n*^EVT^ *n*^PAT^ (%)	0.xxx
Related SAEs	*n*^EVT^ *n*^PAT^ (%)	*n*^EVT^ *n*^PAT^ (%)	0.xxx
Adverse events (AEs)	*n*^EVT^ *n*^PAT^ (%)	*n*^EVT^ *n*^PAT^ (%)	0.xxx
Healthcare utilisation			
Physiotherapy	*n*^EVT^ *n*^PAT^ (%)	*n*^EVT^ *n*^PAT^ (%)	0.xxx
Imaging	*n*^EVT^ *n*^PAT^ (%)	*n*^EVT^ *n*^PAT^ (%)	0.xxx
General practitioner	*n*^EVT^ *n*^PAT^ (%)	*n*^EVT^ *n*^PAT^ (%)	0.xxx
Specialist doctor	*n*^EVT^ *n*^PAT^ (%)	*n*^EVT^ *n*^PAT^ (%)	0.xxx
ED/hospitalisation	*n*^EVT^ *n*^PAT^ (%)	*n*^EVT^ *n*^PAT^ (%)	0.xxx
Others	*n*^EVT^ *n*^PAT^ (%)	*n*^EVT^ *n*^PAT^ (%)	0.xxx
Use of concomitant medications			
Simple analgesia	*n*^EVT^ *n*^PAT^ (%)	*n*^EVT^ *n*^PAT^ (%)	0.xxx
NSAID	*n*^EVT^ *n*^PAT^ (%)	*n*^EVT^ *n*^PAT^ (%)	0.xxx
Combination opioid	*n*^EVT^ *n*^PAT^ (%)	*n*^EVT^ *n*^PAT^ (%)	0.xxx
Strong opioid	*n*^EVT^ *n*^PAT^ (%)	*n*^EVT^ *n*^PAT^ (%)	0.xxx
Others	*n*^EVT^ *n*^PAT^ (%)	*n*^EVT^ *n*^PAT^ (%)	0.xxx
Use of health services and concomitant medicines in patients reporting ongoing pain			
Week 26	*n*/*N* (%)	*n*/*N* (%)	0.xxx
Week 52	*n*/*N* (%)	*n*/*N* (%)	0.xxx
At risk of misuse (scoring ≥9 on COMM)			
Week 12	*n*/*N* (%)	*n*/*N* (%)	0.xxx
Week 26	*n*/*N* (%)	*n*/*N* (%)	0.xxx
Week 52	*n*/*N* (%)	*n*/*N* (%)	0.xxx

*COMM* Current Opioid Misuse Measure

**Table 6:** Detailed adverse events

	Opioid (*n* = *x*)	Placebo (*n* = *x*)
ICD-10 code A	*n*^EVT^ *n*^PAT^ (%)	*n*^EVT^ *n*^PAT^ (%)
ICD-10 code B	*n*^EVT^ *n*^PAT^ (%)	*n*^EVT^ *n*^PAT^ (%)
(etc.)		
Adverse event		
ICD-10 code A	*n*^EVT^ *n*^PAT^ (%)	*n*^EVT^ *n*^PAT^ (%)
ICD-10 code B	*n*^EVT^ *n*^PAT^ (%)	*n*^EVT^ *n*^PAT^ (%)
(etc.)		

## Abbreviations

OPAL: OPioids for acute SpinAL pain
GP: General practitioner
CONSORT: Consolidated Standards of Reporting Trials
SF-12: 12-item Short Form survey (quality of life measurement tool)
ITT: Intention-to-treat
SD: Standard deviation
ID: Identification
CI: Confidence interval
QALY: Quality-adjusted life years
SF-6D: Short Form Six Dimension
ICD-10: International Classification of Disease version 10
COMM: Current Opioid Misuse Measure
BPI: Brief Pain Inventory
RMDQ: Roland-Morris Disability Questionnaire
NDI: Neck Disability Index
SF12v2: Short form 12, version 2
