# Correction: OPAL: a randomised, placebo-controlled trial of opioid analgesia for the reduction of pain severity in people with acute spinal pain—a statistical analysis plan

**DOI:** 10.1186/s13063-023-07525-4

**Published:** 2023-07-27

**Authors:** Caitlin M. P. Jones, Chung-Wei Christine Lin, Richard O. Day, Bart W. Koes, Jane Latimer, Chris G. Maher, Andrew McLachlan, Laurent Billot

**Affiliations:** 1 Institute for Musculoskeletal Health, Sydney Local Health District and The University of Sydney, Level 10N, KGV Building, Missenden Road, Camperdown, Sydney, NSW Australia; 2 Faculty of Pharmacy and The Centre for Education and Research On Ageing (CERA), The University of Sydney and Concord Hospital, Sydney, NSW Australia; 3 Department of Clinical Pharmacology & Toxicology, St Vincent’s Hospital Sydney and St Vincent’s Clinical School, Faculty of Medicine, University of New South Wales, Sydney, NSW Australia; 4 Department of General Practice, Erasmus MC, Rotterdam, The Netherlands; 5 The George Institute for Global Health, Faculty of Medicine, University of New South Wales, Sydney, Australia

**Correction: BMC Trials 23, 212 (2022)**

**https://doi.org/10.1186/s13063-022-06028-y**

Following publication of the original article [1], we have been informed that the name of a supplementary funder was incorrectly reported. “WorkSafe SA” should actually be “ReturnToWorkSA”.

The original article has been corrected.
